# Induction of Immune Tolerance to Foreign Protein via Adeno-Associated Viral Vector Gene Transfer in Mid-Gestation Fetal Sheep

**DOI:** 10.1371/journal.pone.0171132

**Published:** 2017-01-31

**Authors:** Marcus G. Davey, John S. Riley, Abigail Andrews, Alec Tyminski, Maria Limberis, Jennifer E. Pogoriler, Emily Partridge, Aliza Olive, Holly L. Hedrick, Alan W. Flake, William H. Peranteau

**Affiliations:** 1 Center for Fetal Research, Children's Hospital of Philadelphia, Philadelphia, Pennsylvania, United States of America; 2 Gene Therapy Program, Department of Pathology and Laboratory Medicine, University of Pennsylvania, Philadelphia, Pennsylvania, United States of America; 3 Department of Pathology and Laboratory Medicine, Children’s Hospital of Philadelphia, Philadelphia, Pennsylvania, United States of America; University of Nantes, FRANCE

## Abstract

A major limitation to adeno-associated virus (AAV) gene therapy is the generation of host immune responses to viral vector antigens and the transgene product. The ability to induce immune tolerance to foreign protein has the potential to overcome this host immunity. Acquisition and maintenance of tolerance to viral vector antigens and transgene products may also permit repeat administration thereby enhancing therapeutic efficacy. *In utero* gene transfer (IUGT) takes advantage of the immunologic immaturity of the fetus to induce immune tolerance to foreign antigens. In this large animal study, *in utero* administration of AAV6.2, AAV8 and AAV9 expressing green fluorescent protein (GFP) to ~60 day fetal sheep (term: ~150 days) was performed. Transgene expression and postnatal immune tolerance to GFP and viral antigens were assessed. We demonstrate 1) hepatic expression of GFP 1 month following *in utero* administration of AAV6.2.GFP and AAV8.GFP, 2) *in utero* recipients of either AAV6.2.GFP or AAV8.GFP fail to mount an anti-GFP antibody response following postnatal GFP challenge and lack inflammatory cellular infiltrates at the intramuscular site of immunization, 3) a serotype specific anti-AAV neutralizing antibody response is elicited following postnatal challenge of *in utero* recipients of AAV6.2 or AAV8 with the corresponding AAV serotype, and 4) durable hepatic GFP expression was observed up to 6 months after birth in recipients of AAV8.GFP but expression was lost between 1 and 6 months of age in recipients of AAV6.2.GFP. The current study demonstrates, in a preclinical large animal model, the potential of IUGT to achieve host immune tolerance to the viral vector transgene product but also suggests that a single exposure to the vector capsid proteins at the time of IUGT is inadequate to induce tolerance to viral vector antigens.

## Introduction

Adeno-associated viral vectors (AAVs) hold considerable promise for the therapeutic management of a spectrum of life-threatening inherited disorders. AAVs are non-pathogenic and can result in durable expression of the transgene product without incorporating into the host genome making them one of the most clinically relevant viral vector systems. A major limitation to successful AAV gene transfer is the generation of host immune responses to vector capsid proteins and the transgene product [1–3]. Experimentally, the generation of anti-AAV neutralizing antibodies following initial vector exposure has been shown to inhibit transduction upon repeat vector delivery [4,5]. Since repeat administration of AAV vector and the corrective transgene will be necessary for the management of many target diseases, a clinical need exists to develop safe strategies to overcome host immune responses to both the transgene product and the vector capsid proteins.

*In utero* gene transfer (IUGT) is a novel therapeutic strategy that takes advantage of normal developmental ontogeny to induce immune tolerance to the transgene product. During early gestation and prior to thymic processing of mature lymphocytes, the fetal immune system is largely tolerant to foreign antigens [6–10]. Appropriate presentation of foreign antigen, including wild-type transgene product, to the fetal thymus during early development has the potential to induce life-long tolerance to the foreign antigen. Hemophilia B mice deficient in clotting Factor IX (FIX) that underwent IUGT via either intramuscular AAV1 [11] or intraperitoneal (IP) delivery of VSVG-lentivirus vector [12] expressing the FIX transgene demonstrated amelioration of disease and immune tolerance to FIX during postnatal life. Likewise, in pre-immune fetal sheep, IP injection of a retroviral vector resulted in life-long expression of the β-galactosidase (β-gal) transgene product in hematopoietic stem cells and β-gal specific immune tolerance [13]. Finally, in mice, *in utero* exposure to AAV enabled repeat postnatal administration of AAV while avoiding a humoral immune response to vector capsid proteins [14]. The ability of *in utero* delivery of transgene products via the clinically relevant AAV system to induce transgene and vector capsid protein specific immune tolerance in a large animal model warrants investigation.

We recently demonstrated that intra-tracheal delivery of AAV6.2 expressing green fluorescent protein (GFP) to fetal sheep unexpectedly resulted in robust hepatic transduction at three weeks post-injection [15]. In the current study, we evaluate organotropism and durability of GFP transgene expression following IUGT with AAV6.2.GFP, AAV8.GFP, and AAV9.GFP delivered intravenously in the pre-immune fetal sheep. We demonstrate that postnatal injection of GFP in naïve sheep is capable of eliciting a humoral immune response. With this knowledge, recipients of AAV.GFP IUGT were re-challenged postnatally with GFP as well as the same prenatally delivered AAV serotype to determine if IUGT results in immune tolerance to the foreign transgene product and viral vector antigens in this preclinical large animal model.

## Materials and Methods

### Animal use and care

Experimental protocols were approved by the Institutional Animal Care and Use Committee at The Children’s Hospital of Philadelphia (protocol #: 15–000878) and followed guidelines set forth in the National Institute of Health, Guide for the Care and Use of Laboratory Animals. Rigorous methods were undertaken to minimize potential pain and distress of sheep involved in this study. Specifically, for ewes undergoing survival surgery, a fentanyl patch (2mcg/kg/hr) was placed 12–24 hours prior to surgery and removed 72 hours after placement. Buprenorphine (IM; 0.005mg/kg) was administered prior to surgery concurrently with intramuscular Ketamine for initial sedation. A second dose of buprenorphine (IM; 0.005 mg/kg) was administered in the post-operative period, approximately 3–6 hours after the initial dose. Sheep were observed for recovery and signs of distress including elevated respiratory rate, decreased activity level, abnormal body temperature, and evidence of wound infection/dehiscence. Signs of pain/distress were communicated to veterinary staff and an appropriate plan of care developed. At the appropriate time, euthanasia for lambs was provided by Pentobarbital/phenytoin sodium (Euthasol, Beuthanasia-D)(117 mg/kg). A protocol was in place to determine humane endpoints based on the presence of the following clinical signs: the inability to access food and water, labored breathing, the inability maintain an upright/sternal position, paralysis, and gradual weight loss leading to emaciation (animals were weighed at least weakly). If an animal met endpoint criteria, the animal was immediately euthanized. In the current study, however, all animals were euthanized at the planned end of study time point. No animal met the criteria of a humane endpoint nor did any animal die before meeting these criteria for euthanasia.

### AAV vectors

The production of AAV vectors has been previously described [16,17]. Briefly, recombinant AAV flanked with AAV2 inverted terminal repeats (ITRs) contained either enhanced green fluorescent protein (GFP) or firefly luciferase (Luc) under transcriptional control of the cytomegalovirus (CMV) promoter. Recombinant AAV genomes equipped with AAV2 ITRs were packaged by triple transfection of HEK 293 cells with cis-plasmid, adenovirus helper plasmid and a chimeric packaging construct in which the AAV2 rep gene is fused with cap genes of AAV.

### Prenatal AAV screening studies in sheep

Organotropism of AAV6.2.GFP, AAV8.GFP and AAV9.GFP was determined in mid-gestation fetal sheep. Sixty to sixty-five day gestation (term: ~150 days) fetuses were exposed via a hysterotomy and received an intravenous (300–500μL volume) vector injection via the umbilical vein using a 26G needle. Fetuses were sacrificed 4 weeks after injection and transduced organs identified using GFP fluorescence stereomicroscopy and confirmed by GFP immunohistochemistry on formalin-fixed, paraffin-embedded 3–5μm tissue sections as previously described [15]. Organs examined included brain, thymus, heart, lungs, diaphragm, spleen, liver, bowel, kidneys, adrenal glands, hindlimb muscle, umbilical cord and placentae.

### Assessment of immune tolerance following IUGT

**Fig 1:**
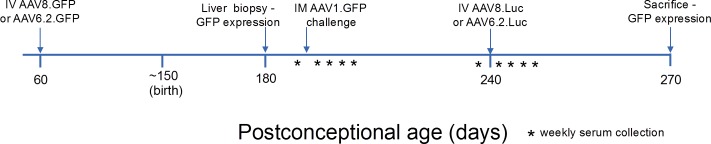
Experimental scheme for assessment of induction of postnatal tolerance by IUGT. IM, intramuscular; IV, intravenous; Luc, luciferase.

We sought to determine if *in utero* administration of AAV.GFP resulted in immune tolerance, as indicated by the lack of an antibody response, to the viral vector capsid proteins or the GFP transgene. Initial studies evaluated the presence of GFP antibodies by ELISA (see ELISA analysis below) in the serum of fetal sheep (used in the vector organotropism studies) four weeks following a single *in utero* injection of AAV6.2.GFP, AAV8.GFP or AAV9.GFP. We also assessed the presence of AAV9 antibodies prior to and 4 weeks after IUGT in the serum of the fetal recipient (from the vector organotropism studies) of AAV9.GFP which failed to demonstrate transduction. We then sought to evaluate immunologic tolerance to the capsid proteins and the GFP transgene in adult life following postnatal antigen challenge using the experimental design depicted in Fig 1. AAV8.GFP and AAV6.2.GFP were intravenously (IV) administered via the umbilical vein to gestational day 60 fetal sheep. At 1 month of age (~120 days post-injection), a liver wedge biopsy (~0.5x0.5x0.25cm) was performed under general inhalation anesthesia. Hepatic GFP expression was assessed by fluorescence stereomicroscopy and GFP immunohistochemistry of formalin-fixed, paraffin-embedded sections (3–5μm) [15]. Characterization of transduced cells based on morphology and anatomical location was performed by an attending pediatric pathologist (J.E.P.). The number of GFP^+^ cells per high power field (HPF; 400X) was calculated based on the evaluation of 20 HPFs per animal. Lambs were subsequently challenged to determine tolerance to GFP and the AAV capsid proteins. Based upon prior murine studies, the body weight-adjusted dose of GFP protein (mg/kg of body weight) for a 10kg lamb was cost-prohibitive. For this reason, lambs were postnatally challenged with GFP using an AAV vector-driven approach via a hindlimb (tibialis anterior) intramuscular (IM) injection of AAV1.CMV.GFP [400μL; 1.10x10^12^ genome copies (GC)], an AAV serotype previously shown to transduce striated muscle [11,16]. As control, a 3 month old lamb that did not receive an *in utero* injection of AAV.GFP was injected IM with AAV1.GFP. To evaluate tolerance to AAV capsid proteins, lambs were challenged at 12 weeks of life with the same vector serotype they received during fetal life, namely AAV6.2 (4.01x10^11^ GC) or AAV8 (2.30x10^11^ GC) expressing firefly luciferase delivered IV (400μL). This AAV challenge was delivered IV to mimic potential clinical scenarios in which repeat IV doses of viral vectors with a therapeutic transgene are required to achieve/maintain a therapeutic effect. The AAV challenge vector expressed firefly luciferase to avoid influencing ongoing studies of durability of hepatic GFP expression in these animals. Blood samples (10mL) were collected prior to vector challenge and at weekly intervals for 4 weeks post challenge. Samples were centrifuged (900g, 15mins, 4°C) and serum stored at -80°C. Serum antibodies against GFP or capsid antibodies were measured by ELISA as described below.

### ELISA analysis of serum GFP and vector capsid protein antibodies

Non-tissue culture treated 96-well plates (CytoOne, Ocala, FL, USA; Catalog# CC7672-7596) were coated with 100μL per well of recombinant GFP (5μg/mL; Vector Laboratories, Burlingame, CA, USA; Catalog# MB-0752) or heated-inactivated (90°C for 30 mins) AAV (AAV6.2: 4.01x10^9^ GC per well; AAV8: 2.30x10^9^ GC per well; AAV9: 7.5x10^10^ GC per well) and incubated overnight at 4°C. Plates were washed three times with room temperature PBS and incubated with blocking buffer (Catalog# MB-070, 100uL per well; Rockland Immunochemicals, Inc. Gilbertsville, PA, USA) overnight at 4°C. Serum samples were diluted in blocking buffer (100uL volume), loaded into wells as serial dilutions from 1:50 to 1:6400 and incubated overnight at 4°C. Plates were subsequently washed with PBS/0.1% Tween20 and PBS. Donkey anti-sheep IgG horseradish peroxidase (HRP)-conjugated antibody (Sigma Aldrich, St. Louis, Missouri, USA; Catalog# A3415) diluted to 1:10,000 in blocking buffer (100uL) was loaded into wells and plates incubated for 2 hours at room temperature and then washed each with PBS/0.1% Tween20 followed by PBS alone. HRP was detected with a chromogenic substrate kit according the manufacturer’s instructions (Thermo Fisher Scientific, Hudson, New Hampshire, USA; Catalog# 34021) using an absorbance wavelength of 450nm (Biorad, Hercules, California, USA; Catalog# 168–1135).

### AAV neutralizing antibody assay

AAV6.2 and 8 serum neutralizing antibody (Nab) titers in recipients of IUGT of the corresponding AAV serotype were determined as previously described [17]. Briefly, the reporter virus AAV.CMV.Luc of each serotype was pre-incubated with 2-fold serially diluted heat-inactivated serum collected from lambs prior to postnatal AAV challenge and 4 weeks post-challenge. After 1 hour of incubation at 37°C, the serum/reporter AAV vector mixture was added to Huh7 cells seeded in 96-well plates at a multiplicity of infection equal to 10^4^ and incubated for 24 hours. The assay was developed using luciferin and the signal read by a luminometer. NAb titers are reported as the highest serum dilution at which transduction was reduced by 50% compared to virus control wells (serum with no NAb). The limit of detection of this assay is 1/5 serum dilution.

### Durability of transgene expression

Lambs were euthanized at ~6 months of life. GFP transgene expression was examined in all major organs including brain, thymus, heart, lungs, diaphragm, spleen, liver, bowel, kidneys, and adrenal glands, as well as the site of postnatal challenge (hindlimb muscle) using GFP fluorescence stereomicroscopy and immunohistochemistry on formalin-fixed, paraffin-embedded 3–5μm tissue sections [15]. As indicated above, characterization of transduced liver cells and quantification of the number of GFP^+^ cells per HPF based on the analysis of 20 HPFs per animal was performed by an attending pediatric pathologist (J.E.P.).

## Results

### Prenatal AAV screening studies in sheep

Sixty day fetal sheep were injected with either AAV6.2, AAV8 or AAV9 expressing GFP. These viral vectors were chosen based on our own preliminary studies in the mouse model of IUGT as well as published studies in mouse and large animal models which demonstrated transduction of the liver and other organs by these AAV vector serotypes [18–21]. Organs were harvested one month post injection and assessed for GFP expression (S1 Table, S1 Fig). AAV6.2 and AAV8 demonstrated diffuse hepatic expression of GFP at a low frequency but high signal intensity. GFP expression was absent from all other major fetal organs as well as in the umbilical and placental tissues. The fetal recipient of AAV9 did not demonstrate GFP expression in the placental or umbilical tissue as well as in all other screened organs.

### Induction of immune tolerance to the transgene product (GFP) via IUGT

**Fig 2:**
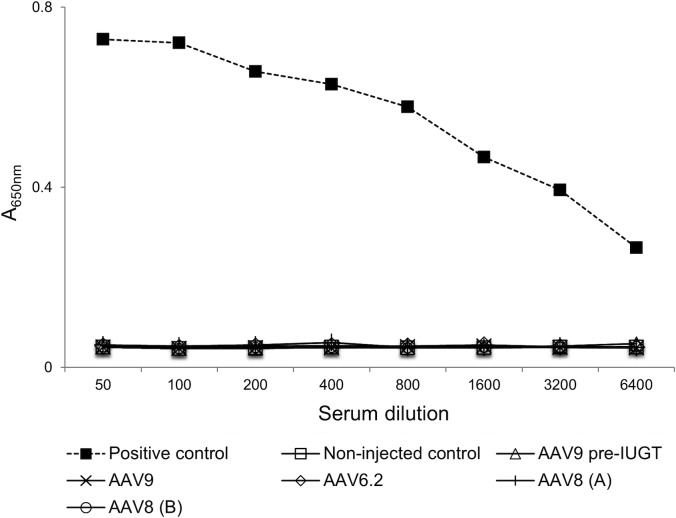
Assessment of GFP transgene specific antibodies at 1 month following IUGT. Serum from fetal recipients of IUGT was assessed at 1 month post injection for the presence of GFP specific antibodies by ELISA. Positive control = serum from a neonatal lamb three weeks after receiving an intramuscular injection of AAV1.GFP; Non-injected control = serum from an aged matched fetus that did not undergo IUGT; AAV9 pre-IUGT = serum from the fetal recipient of AAV9 prior to IUGT; AAV8 (A) and AAV8 (B) refer to samples from 2 different fetuses injected with AAV8.GFP.

Initially we sought to determine if a single *in utero* injection at midgestation results in the production of antibodies specific for the transgene product at early time points following IUGT. Serum was assessed at one month post IUGT for the presence of GFP specific antibodies in those animals used for vector screening studies (Fig 2). No GFP specific antibodies were detected in the recipients of AAV6.2.GFP and AAV8.GFP. Additionally, no serum antibodies against GFP or the AAV vector (S2 Fig) were present in the recipient of AAV9.GFP, which failed to demonstrate GFP expression on screening studies, suggesting that the lack of expression of the AAV9 serotype is secondary to transduction inefficiency as opposed to an immune response against the transgene product or viral vector.

**Fig 3:**
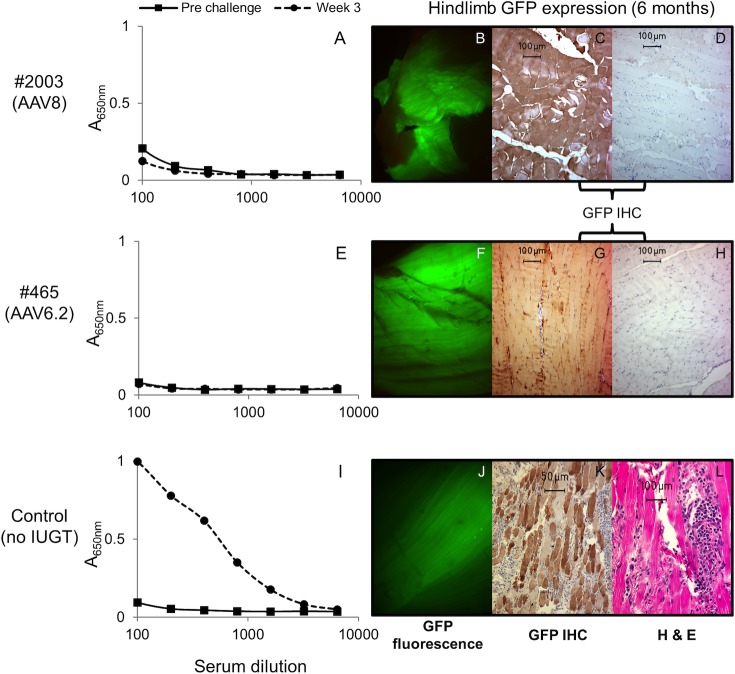
Lack of a postnatal humoral immune response to the GFP transgene product following IUGT. Fetal recipients of AAV8.GFP or AAV6.2.GFP underwent postnatal IM challenge with AAV1.GFP. Serum anti-GFP antibodies prior to challenge and weekly for 4 weeks post challenge were assessed by ELISA. Control animals received the postnatal challenge without IUGT. The control animal generated a robust humoral (IgG) immune response to GFP (I). In contrast, recipients of IUGT with AAV8.GFP (A) or AAV6.2.GFP (E) did not generate anti-GFP antibodies. Results from 3 weeks post challenge are shown. Similar results were seen on analysis at 1, 2 and 4 weeks (data not shown). At 6 months of age, GFP expression was assessed at the site of postnatal challenge in the hindlimb muscle; AAV8 (B,C,D), AAV6.2 (F,G,H) and control (J,K,L). All animals demonstrated GFP expression which was associated with a significant inflammatory cell influx in the control but not the recipients of IUGT. IHC, immunohistochemistry; H&E, hematoxylin and eosin.

We next sought to assess long-term postnatal tolerance induction to the transgene product (GFP) secondary to IUGT by postnatal challenge with GFP. As discussed above, AAV6.2 and AAV8 demonstrated hepatic GFP expression 4 weeks post IUGT and thus were used to assess postnatal tolerance induction. Midgestation fetal recipients of AAV.GFP IUGT underwent a liver biopsy at 1 month of age to confirm transgene expression followed by intramuscular challenge with AAV1.GFP (Table 1, Fig 1). The presence of serum anti-GFP antibodies was assessed prior to challenge and weekly for 4 weeks post challenge. The control lamb received only the postnatal challenge without having previously undergone IUGT. Recipients of IUGT did not demonstrate anti-GFP antibodies at 1 month of age prior to challenge and, in contrast to the control which demonstrated anti-GFP IgG antibodies at weeks 1 through 4 post challenge, failed to mount an anti-GFP antibody response following postnatal challenge at all time points assessed (Fig 3). At the time of sacrifice (6 months of age), GFP was visualized with the unaided eye in the tibialis anterior (the site of postnatal challenge) in the experimental but not control animals. Histologic and fluorescent stereomicroscopic analysis of control and experimental animals demonstrated GFP expression in the tibialis anterior in all animals. This expression was associated with a localized inflammatory cell influx in the control animal and no inflammatory cell response in the experimental animals (Fig 3).

**Table 1:** Postnatal tolerance induction following AAV.GFP IUGT.

Animal #	GA @ IUGT (days)	AAV serotype	Concentration (GC)	GA @ birth (days)	Hepatic GFP expression
2003	64	8	8.69x10^11^	144	Yes
465	62	6.2	1.64x10^11^	146	Yes

GA, gestational age; IUGT, *in utero* gene transfer; GC, genome copies; Hepatic GFP expression assessed at 1 month postnatal age prior to postnatal GFP and AAV vector challenge.

### Immune tolerance to vector capsid proteins of AAV

**Fig 4:**
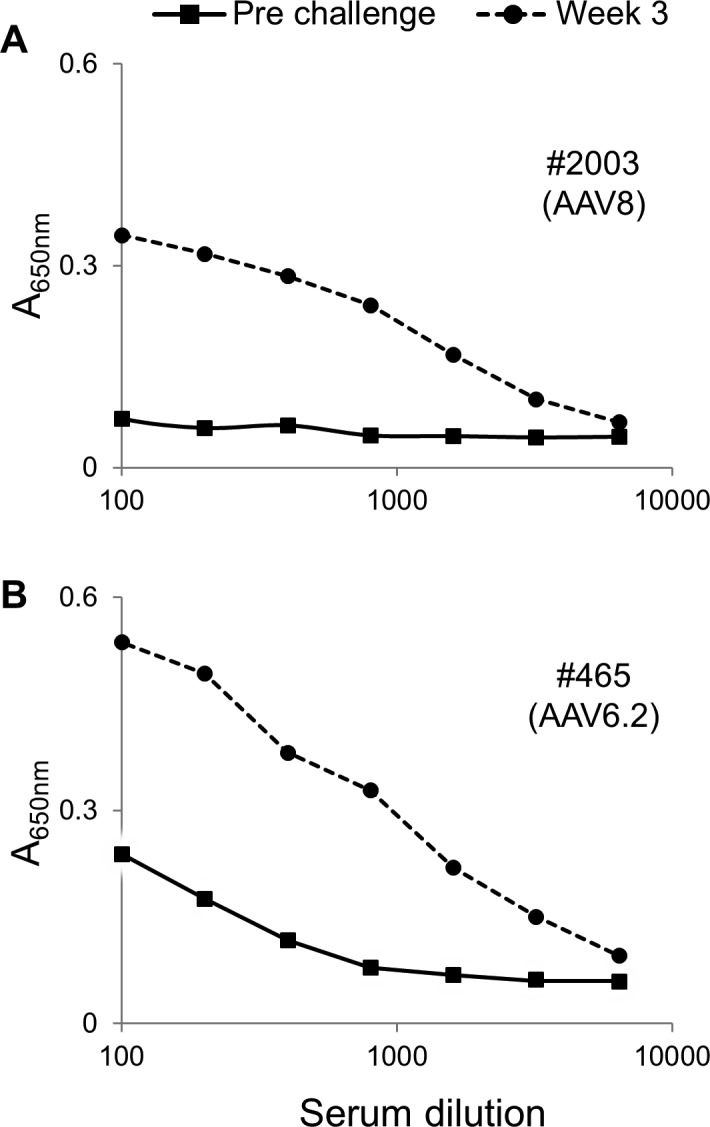
IUGT does not induce humoral immune tolerance to the viral vector capsid proteins. Fetal recipients of AAV8.GFP (A) or AAV6.2.GFP (B) underwent postnatal challenge with AAV8.Luc or AAV6.2.Luc respectively at 3 months of age. The presence of IgG anti-AAV capsid protein antibodies in the serum was assessed weekly for 4 weeks post challenge. An anti-AAV capsid protein antibody response elicited at 3 weeks post challenge is demonstrated. Similar results were seen at 1, 2 and 4 weeks post challenge (data not shown).

We evaluated whether a single IUGT with AAV.GFP can induce immune tolerance to the AAV capsid proteins. As shown in Fig 4, postnatal challenge via intravascular injection of AAV8.Luc or AAV6.2.Luc of recipients of the same AAV serotype expressing GFP delivered *in utero* elicited an anti-AAV serotype specific antibody response. The serum-circulating antibodies were neutralizing with levels of AAV8 and AAV6.2 specific neutralizing antibodies of 1:10 and 1:640 pre-challenge, and 1:2560 and 1:1280 at week 4 post-challenge respectively.

### Durability of transgene expression

**Fig 5:**
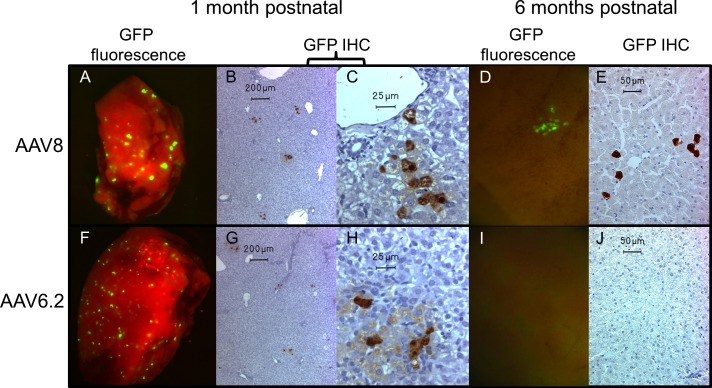
Long-term hepatic GFP expression in lambs undergoing IUGT. Hepatic expression of the GFP transgene was assessed serially in lambs undergoing IUGT with either AAV8.GFP or AAV6.2.GFP. Persistent, but decreased, expression at 6 months of age was seen in the recipient of AAV8 (1 month A,B,C; 6 months D,E). The recipient of AAV6.2 lost hepatic expression from 1 to 6 months of age (1 month F,G,H; 6 months I,J). IHC; immunohistochemistry.

The duration of GFP expression was monitored by liver biopsy at 1 month of age followed by necropsy and screening of all organs at 6 months of age (Fig 5). We observed GFP expression in the liver at 1 month of life (~15 weeks after *in utero* intravenous injection) in recipients of AAV6.2.GFP and AAV8.GFP IUGT. Characterization of the GFP^+^ cells by morphology demonstrated that transduced cells were hepatocytes in recipients of AAV6.2.GFP and AAV8.GFP. The recipient of AAV8.GFP also rarely demonstrated transduction of bile duct epithelial cells (1–2 total cells seen in all samples). Furthermore, quantification of GFP^+^ hepatocytes at 1 month of age suggested that the efficiency of transduction by both vectors was low with a trend toward increased transduction by AAV8 (AAV8.GFP vs. AAV6.2.GFP: 4.1 vs. 3 GFP^+^ cells / HPF). AAV8 is known to be hepatotropic and GFP^+^ hepatocytes were seen in the recipient of AAV8.GFP at 6 months of age although at a significantly decreased frequency compared to 1 month of age (1 month vs. 6 months of age: 4.1 vs. 0.35 GFP^+^ cells / HPF). GFP expression was not seen in any other organs harvested from this animal. Interestingly, AAV6.2 mediated GFP hepatic expression was short-lived and was absent in the liver and all other organs examined at 6 months of age. Similar to the absence of an inflammatory cell infiltrate at the site of postnatal intramuscular challenge (Fig 3), neither a diffuse inflammatory cell infiltrate nor the presence of inflammatory cells around GFP^+^ cells was noted in the liver specimens of recipients of AAV6.2.GFP or AAV9.GFP at the time of biopsy (1 month of age) and the time of sacrifice (6 months of age) (Fig 5 and S3 Fig). No GFP expression was seen in the organs of the control animal which only underwent postnatal IM AAV1.GFP injection. All experimental and control animals expressed varying levels of GFP in the tibialis anterior (the site of postnatal challenge) at the time of sacrifice (Fig 3).

## Discussion

AAV vectors have been investigated in several clinical trials for a spectrum of monogenetic disorders [22–32]. In these trials, a significant barrier to success is the host immune response to the vector capsid proteins and/or the transgene product. This is exemplified in all preclinical and clinical studies of gene therapy for congenital myopathies in which anti-capsid and anti-transgene product related humoral and cellular responses have limited success [23]. As the promise of AAV-based gene therapy becomes increasingly realized, safe strategies to overcome both preexisting and induced host immune responses to vector capsid antigens and the transgene product are required. This challenge is even more significant for genetic diseases which may require repeat administration of the viral vector to boost transgene expression. A number of these genetic diseases such as cystic fibrosis and Duchenne muscular dystrophy can be prenatally diagnosed [33]. The ability to prenatally diagnose a target disease combined with the natural immunologic immaturity of the fetus highlights the possibility of delivering the viral vector and transgene product to a pre-immune fetus with subsequent induction of immune tolerance to vector capsid antigens and the transgene product. In the current study, we demonstrate, in a large animal preclinical model, that postnatal tolerance to a foreign protein (i.e. GFP) can be achieved following intravascular delivery of AAV-IUGT expressing a foreign protein. Despite the ability to induce tolerance to the transgene product, IUGT with AAV.GFP failed to induce tolerance to the AAV vector capsid proteins. Finally, at the termination of the experiment (6 months of age), persistent hepatic transgene expression was seen in the *in utero* recipient of AAV8.GFP while the recipient of AAV6.2.GFP lost transgene expression between 1 and 6 months of age.

The ability of IUGT to induce immune tolerance to the transgene product has been investigated previously. In the murine model, IUGT has been shown to result in tolerance to human factor IX in normal and hemophilia B mice following delivery of the adenoviral, AAV and retroviral vector systems [11,12,34]. Additional mouse studies demonstrated the ability to induce transgene immune tolerance following neonatal gene therapy, an event unlikely to be achieved clinically, thus highlighting the need to study vector- and transgene-associated immune responses in a large animal preclinical model [11,35]. Life-long tolerance to the transgene product has been demonstrated in the sheep model following IUGT with retroviral vectors [13,36]. In those studies, animals that received IUGT failed to mount a humoral response against the transgene product (β-galactosidase) following repeated postnatal immunization whereas there was a robust anti-β-gal antibody response in control animals [36]. Retroviral vectors integrate into the host genome and, if able to target the abundant and expanding stem cell population during fetal life, have the capacity to permanently correct genetic disease [37–39]. However, integrating vectors have an increased risk of vector-associated oncogenesis/tumorogensis [40,41] making them less optimal candidates for clinical trials. Recently, in the sheep model of IUGT, immune tolerance to the transgene product was not achieved following intraperitoneal (IP) administration of AAV8 expressing the factor IX (FIX) transgene [42]. The results of this study may differ from our current study in which postnatal immunization did not elicit an immune response because of the different routes of injection (IP vs. IV) or, alternatively, the different transgene products. Studies of *in utero* hematopoietic cell transplantation (IUHCT) in the fetal canine and mouse models support improved delivery of the injectate to the fetal liver following IV compared to IP injection [43,44]. Similarly, studies of IUHCT in these models also suggest that the ability to induce immune tolerance to an alloantigen is dependent on achieving a threshold level of antigen expression [45,46]. Thus, the inability to efficiently deliver a high enough viral load to the fetal liver following IP IUGT may result in suboptimal induction of tolerance that may be broken following postnatal immunization.

Natural exposure to AAV is frequent early in life resulting in the production antibodies to vector capsid proteins. These naturally occurring antibodies may critically influence the ability to use AAV as a gene therapy vector [2,47]. A potential benefit of IUGT is the introduction of the initial AAV vector prior to the development of natural AAV antibodies postnatally or prior to the potential transfer of naturally occurring AAV antibodies from the mother to the fetus across the placenta. Maternal transfer of IgG across the placenta begins after 14 weeks gestation and peaks in the third trimester in humans [48]. Thus, the ideal time for IUGT would be between 12 and 14 weeks gestation—a time of immunologic immaturity when immune tolerance induction is feasible, prior to maternal transfer of naturally occurring antibodies, and when it is technically feasible to perform an intravascular injection via fetoscopic or ultrasound-guided umbilical vein or intracardiac injection [44]. However, even if one avoids an anti-AAV antibody response from maternally transferred antibodies by performing IUGT at the end of the first trimester, avoiding the postnatal immune response to viral antigens would still be necessary to allow for successful repeat courses of AAV gene therapy to augment levels of the transgene product. Anti-capsid antibodies and AAV capsid specific memory T cells are proposed to compromise efficient repeat transduction with the same vector both shortly after initial AAV gene therapy as well as later in life [23,49]. Unlike immune privileged sites, host-immune responses to AAV have been reported following intrahepatic [28], intramuscular [50] and intra-airway [51] vector delivery. In the current study, a single IUGT with AAV.GFP failed to induce immune tolerance to the vector capsid proteins. This is in contrast to studies in the murine model in which *in utero* or neonatal gene transfer resulted in reduced levels or absence of neutralizing antibody responses to viral antigens following postnatal challenge [14,52]. Our results, in a large animal model, suggest that sustained expression of AAV capsid antigens during thymic development, and possibly throughout the lifetime of the individual, may be necessary to achieve tolerance to viral antigens. As such, alternative mechanisms to overcome the immune response to viral antigens including B cell depletion via Rituximab and Sirolimus prior to AAV administration, the use of empty capsids or capsid decoys to “absorb” antibodies, and single amino acid modification of viral capsid proteins are being explored and hold clinical promise [23,53,54].

We demonstrated hepatic GFP expression in postnatal lambs at 1 month of age following IUGT with AAV6.2 and AAV8. AAV8, which is known to be hepatotropic, demonstrated a trend toward increased transduction efficiency compared to AAV6.2 and the GFP^+^ cells persisted at 6 months of age in the recipient of AAV8.GFP. This finding is consistent with previous studies in the postnatal canine model demonstrating improved hepatic transduction with AAV8 vs. AAV6.2 [18]. The initial inefficient hepatocyte transduction by AAV6.2 compared to AAV8, combined with physiologic growth and apoptosis of transduced cells from the time of in utero delivery to 6 months of age likely resulted in the loss of GFP^+^ cells between 1 and 6 months of age in recipients of AAV6.2. It is important to note that, although GFP^+^ cells were detected at 6 months of age in the recipient of AAV8.GFP, the number of transduced cells at this time was significantly reduced compared to the 1 month time point and that continued physiologic growth and hepatocyte turn-over may result in loss of GFP^+^ hepatocytes in this animal as well. However, hepatic GFP expression in postnatal lambs at 6 months of age (~9 month after in utero vector administration) following in utero AAV8.GFP administration was an unexpected finding in light of a recent report of AAV-IUGT in sheep. David and colleagues performed IP injections in 60–65 day gestation fetal sheep of AAV8 (1x10^12^ GC/kg) expressing a secretable human FIX (hFIX) under a liver specific promoter [42]. In that study, transgene product (i.e. serum levels of hFIX) declined during *in utero* development and, by 1 month after birth, plasma hFIX was undetectable; at 1 year of life, hepatic expression of AAV8 transgene was absent. A decline in AAV transgene expression following IUGT was also observed in mice [55] and is likely a consequence of the organ growth and normal apoptosis of transduced cells. As a point of reference, the body weight of sheep increases ~450 fold over the course of our study from ~100 grams at 65 days of gestation [54], to ~10kg at one month of age (time of liver biopsy), and ~45kg at six months of age. Similarly, the liver weight of sheep increases from ~15 grams at 80 days gestation to ~251 grams at 1 month of age and ~507 grams at 6 months of age representing an increase of at least 34 fold from the time of in utero injection to analysis at 6 months of age [56]. Following IUGT with AAV8, hepatocyte transgene expression, as determined by the density of GFP positive cells per visual microscopic field, decreased with the growth of our lambs but did persist for up to 6 months of age. The durable expression in our study, unlike that observed in the previous AAV-IUGT sheep study, may be a consequence of the route of vector delivery (IP versus IV; IV resulting in more efficient initial transduction of hepatocytes as discussed above), promoter (LP-1 versus CMV), or fetal immune response (or lack thereof) to the transgene product (hFIX vs. GFP). Regarding the later, it is proposed that a “window of opportunity” exists in early development, between 45–65 days gestation in sheep, during which the fetus can be rendered immunologically tolerant to foreign antigens. However, this window and the receptivity of the fetal sheep to foreign antigen may differ depending on the type of antigen. This premise is supported by studies demonstrating tolerance to skin homografts and cellular/genetic therapies performed prior to 71 days gestation [8,13,57,58] while the bacteriophage virus ØX174 stimulates antibody formation when injected at 35 days of gestation [7]. Finally, in the current study, persistent hepatic GFP expression at 6 months of age is presumed to originate from the IUGT. However, these animals received a postnatal challenge with AAV1.GFP via the IM route at 1 month of age. Thus, we cannot exclude the possibility that hepatocyte GFP expression in experimental animals resulted not only from IUGT but also from the postnatal AAV1.GFP IM challenge. In this scenario, the lack of hepatocyte GFP expression in the control animal, which only received a postnatal AAV1.GFP IM challenge, is explained by an immune response to the transgene.

Another interesting finding in our current study was the lack of transduction of any organ, including the liver, following in utero delivery of AAV9.GFP. We chose to study AAV9 as well as AAV8 and AAV6.2 based on our own preliminary data in the mouse model as well as previously published studies in mouse and large animal models demonstrating that these serotypes transduced hepatocytes [18–21]. Potential explanations for the absence of GFP^+^ cells in the liver following in utero AAV9.GFP delivery include: 1. inefficient transduction secondary to the absence or low levels of the serotype’s ligand on the fetal liver at the time of injection, 2. a poor quality, nonfunctioning AAV9 vector, 3. the presence of pre-existing naturally occurring anti-AAV9 antibodies in the fetal sheep (presumable via placental transfer of maternal antibodies following natural exposure), or 4. the induction of an immune response to the GFP transgene product. Analysis of the serum of the recipient of AAV9.GFP four weeks post IUGT did not demonstrate the presence of anti-GFP antibodies suggesting that the latter possibility is not the cause. Naturally occurring immunity to the AAV9 serotype has been demonstrated in postnatal sheep [59]. However, unlike humans, in which maternal IgG can cross the placenta into the fetus [48], maternal antibodies are not transferred across the placenta in sheep [60,61]. These studies, together with our inability to detect anti-AAV9 antibodies in the fetal serum prior to or after AAV9.GFP IUGT, make it unlikely that anti-AAV9 antibodies prevented AAV9.GFP transduction. Finally, the AAV9.GFP vector used in the current study has been demonstrated to successfully transduce mouse hepatocytes following in utero delivery ([19] and preliminary data not shown) and pulmonary epithelial cells in 1 week old sheep following endobronchial delivery [62] confirming that the vector is of good quality and capable of transducing cells. Thus, we believe, that the absence of GFP^+^ hepatocytes following in utero AAV9.GFP delivery in the current study is secondary to the absence or low levels of the serotype’s ligand on the sheep fetal liver at the time of injection.

We acknowledge the caveats of the current study. Firstly, the small number of animals per group prevents rigorous statistical comparisons and is a consequence of the high maintenance costs associated with the large animal ovine model. While our data should be interpreted with caution, due to few experimental animals, the humoral and histological data support the premise that immune tolerance can be achieved via intravascular AAV-IUGT. Secondly, although we demonstrate a lack of anti-GFP antibodies in fetal sheep at 1 month post-injection (Fig 2), these data include pre-injection reference samples from only one of the injected fetuses (the recipient of AAV9.GFP) in an attempt to minimize the risk of fetal loss prior to IUGT in the other fetuses. The analysis of an age matched fetus not undergoing IUGT, however, was performed to provide an additional reference. Additionally, there was no histologic evidence of a cellular immune response in the muscle and/or livers of fetal sheep at 1 month post-injection further supporting the absence of a GFP-mediated immune response. Thirdly, although we demonstrate the lack of a humoral immune response to the transgene product following postnatal challenge in recipients of IUGT and also demonstrate no inflammatory cell infiltrate at the intramuscular challenge site or liver at the time of biopsy and sacrifice, additional studies are warranted to support the finding that IUGT induces cellular immune tolerance as well as humoral immune tolerance to the transgene product. Lastly, examining long-term tolerance in the lamb which lost hepatic GFP expression between 1 and 6 months after birth may elucidate whether “maintenance of the tolerant state depend[s] upon the continuing presence of the antigen that provoked it?” [63]. The loss of GFP expression in this animal was realized at post-mortem and hence additional studies would be necessary to address this question.

In summary, we demonstrate the ability to achieve low-level hepatic transgene expression at 6 months of age following in utero delivery of AAV8.GFP in a large animal, preclinical model. In utero intravenous delivery of AAV8.GFP and AAV6.2.GFP at 60–65 days gestation is associated with the absence of a humoral response to the transgene product following postnatal challenge but the ability to elicit an immune response to the viral vector capsid proteins remains. These findings advance those of the previous study by David et al. [42], which demonstrated more transient expression of hFIX and the inability to induce immune tolerance to the hFIX transgene product following in utero intraperitoneal AAV8.hFIX injection in fetal sheep. Together, these studies, suggest that efficient delivery of a vector transgene to the fetal liver, potentially via an intravenous route, can result in successful immune tolerance induction to the transgene product. However, they highlight the need for alternative approaches to achieve immune tolerance to viral vector capsid proteins, the acquisition of which will be important to treat diseases requiring repeated postnatal administration of the therapeutic viral vector.

## Supporting Information

S1 Fig Organotropism of AAV serotypes in fetal sheep following IUGT. AAV6.2, AAV8 and AAV9 expressing the GFP transgene were injected via the umbilical vein into fetal sheep at the concentration indicated in S1 Table. Organs were assessed by GFP immunohistochemistry and stereomicroscopy 1 month post-injection. Representative images of hepatic transduction by AAV6.2 (A,B) and AAV8 (C,D) are shown.
(TIFF)

S2 Fig Absence of anti-AAV9 antibodies in the serum of the fetal recipient of AAV9.GFP. Serum from the fetal recipient of AAV9.GFP (Animal #2156) was obtained immediately prior to IUGT with AAV9.GFP and 1 month following IUGT. The presence of AAV9 specific antibodies was assessed by ELISA. The negative control consists of wells in which only the secondary antibody was added thus providing background fluorescence and is represented as the average absorbance of the 4 control wells since no primary serum dilutions were performed.
(TIFF)

S3 Fig Assessment of hepatic inflammatory infiltrates following IUGT. Liver specimens were obtained from fetal recipients of AAV8.GFP or AAV6.2.GFP at the time of biopsy at 1 month of age and at the time of sacrifice at 6 months of age. Specimens were processed and stained with hematoxylin and eosin and assessed by histology for inflammatory cell infiltrates. Representative images from the fetal recipient of AAV8 (animal #2003) at 1 month (A, 10x; B, 40x) and 6 months (C, 10x; B, 40x) of age as well as the fetal recipient of AAV6.2 (animal #465) at 1 month (E, 10x; F, 40x) and 6 months (G, 10x; H, 40x) of age are shown.
(TIFF)

S1 Table Organotropism of AAV serotypes in fetal sheep following IUGT. GA, gestational age; IUGT, *in utero* gene transfer; GC, genome copies.
(TIFF)
