# Supplementary figures and images for: Induction of Immune Tolerance to Foreign Protein via Adeno-Associated Viral Vector Gene Transfer in Mid-Gestation Fetal Sheep
Source: PLoS One. 2017 Jan 31;12(1):e0171132. doi: 10.1371/journal.pone.0171132 (PMC5283730; doi:10.1371/journal.pone.0171132)

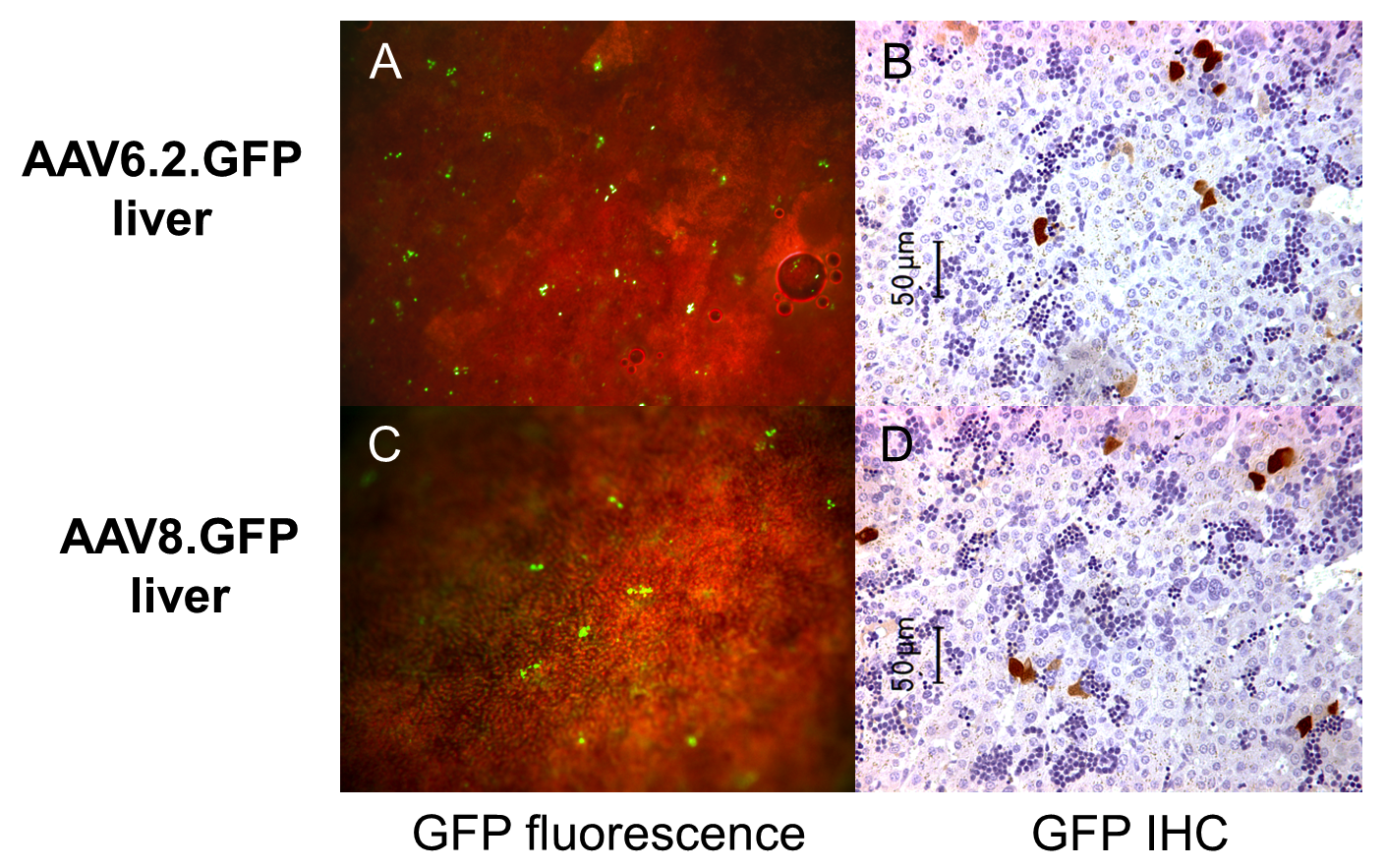

Supplement: S1 Fig — AAV6.2, AAV8 and AAV9 expressing the GFP transgene were injected via the umbilical vein into fetal sheep at the concentration indicated in S1 Table. Organs were assessed by GFP immunohistochemistry and stereomicroscopy 1 month post-injection. Representative images of hepatic transduction by AAV6.2 (A,B) and AAV8 (C,D) are shown. (TIFF) [file pone.0171132.s001.tiff]

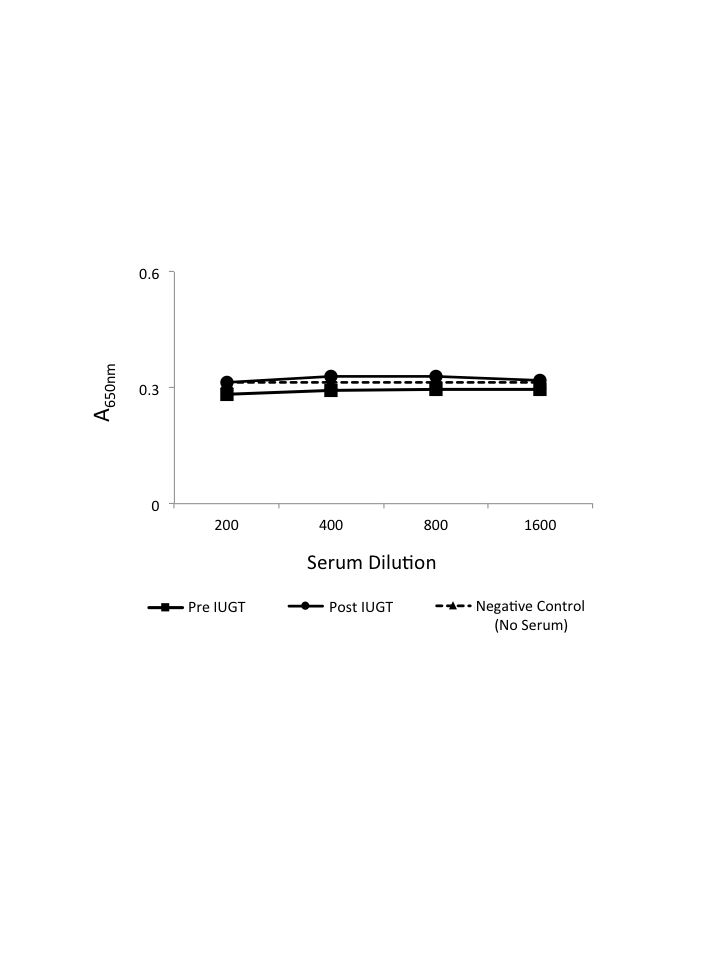

Supplement: S2 Fig — Serum from the fetal recipient of AAV9.GFP (Animal #2156) was obtained immediately prior to IUGT with AAV9.GFP and 1 month following IUGT. The presence of AAV9 specific antibodies was assessed by ELISA. The negative control consists of wells in which only the secondary antibody was added thus providing background fluorescence and is represented as the average absorbance of the 4 control wells since no primary serum dilutions were performed. (TIFF) [file pone.0171132.s002.tiff]

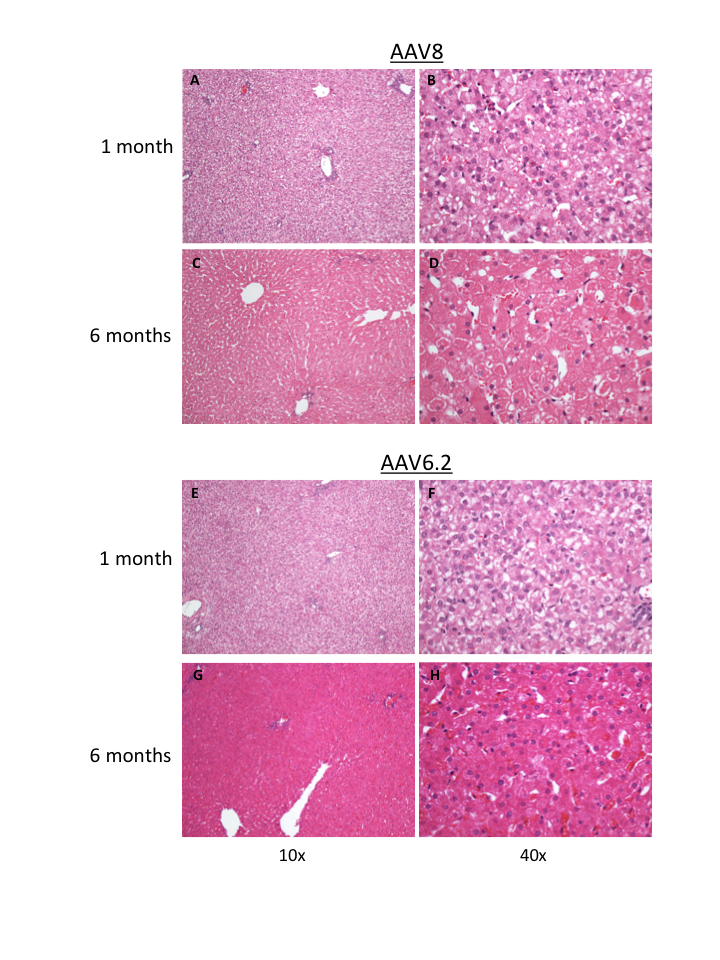

Supplement: S3 Fig — Liver specimens were obtained from fetal recipients of AAV8.GFP or AAV6.2.GFP at the time of biopsy at 1 month of age and at the time of sacrifice at 6 months of age. Specimens were processed and stained with hematoxylin and eosin and assessed by histology for inflammatory cell infiltrates. Representative images from the fetal recipient of AAV8 (animal #2003) at 1 month (A, 10x; B, 40x) and 6 months (C, 10x; B, 40x) of age as well as the fetal recipient of AAV6.2 (animal #465) at 1 month (E, 10x; F, 40x) and 6 months (G, 10x; H, 40x) of age are shown. (TIFF) [file pone.0171132.s003.tiff]

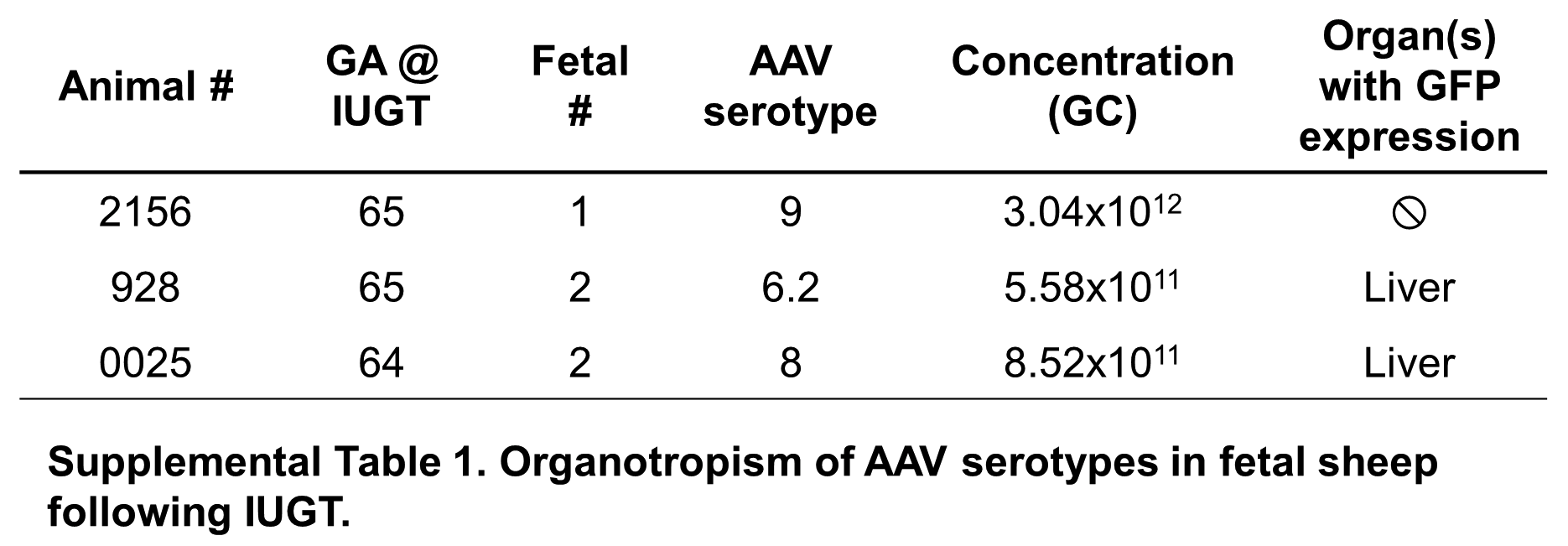

Supplement: S1 Table — GA, gestational age; IUGT, in utero gene transfer; GC, genome copies. (TIFF) [file pone.0171132.s004.tiff]
